# Drug‐induced lung injury in a patient treated with prior atezolizumab and subsequent sotorasib: A case report

**DOI:** 10.1002/rcr2.1284

**Published:** 2024-01-24

**Authors:** Yuta Takahashi, Hiroaki Tachi, Ryo Watanabe, Kei Shimizu, Yusuke Yamamoto

**Affiliations:** ^1^ Department of Respiratory Medicine Hitachi General Hospital, Hitachi Ltd. Hitachi City Japan

**Keywords:** clinical diagnosis, drug‐induced lung injury, immune checkpoint inhibitor, response to prednisolone, sotorasib

## Abstract

We report a case of drug‐induced lung injury treated with prior atezolizumab and subsequent sotorasib. The patient was a 62‐year‐old woman with lung adenocarcinoma harbouring a KRAS G12C mutation that was resistant to chemotherapy, including immune checkpoint inhibitors. Cough and dyspnea appeared on day 80 after sotorasib was administered as second‐line therapy, and chest computed tomography revealed ground glass opacities in all lung lobes. Bronchoalveolar lavage fluid showed an increased total cell count with lymphocyte predominance. The patient was considered to have lung injury caused by prior atezolizumab or sotorasib administration. Withdrawal of sotorasib did not improve symptoms and shadows in both lungs. We administered moderate‐dose prednisolone and the lung disorder quickly resolved. Prednisolone tapering was completed in 2 months, followed by several months without relapse. Definitive identification of the responsible drug for the drug‐induced lung injury proved challenging in the setting of exposure to multiple potential inciting agents. There is a need for high levels of clinical suspicion for timely evaluation and management.

## Graphical abstract

We report a case of drug‐induced lung injury treated with prior atezolizumab and subsequent sotorasib. Definitive identification of the responsible drug for the drug‐induced lung injury proved challenging in the setting of exposure to multiple potential inciting agents. There is a need for high levels of clinical suspicion for timely evaluation and management.

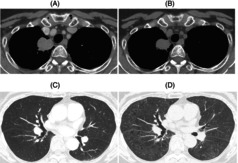

## INTRODUCTION

The diagnosis of drug‐induced lung injury is often made as a diagnosis of exclusion and rarely requires a histological basis for its diagnosis. In the presence of multiple associated drugs, the clinical course is often used to identify the suspected drug. Sotorasib is a molecularly targeted agent administered in second‐line therapy for advanced non‐small cell lung cancer, usually with an immune checkpoint inhibitor (ICI) as pre‐treatment. No lung adverse events attributed to sotorasib have been reported. The absence of reports detailing sotorasib‐induced lung injury may stem from challenges in differentiating it from lung injuries caused by prior ICIs, possibly leading to an underestimation of its actual incidence in clinical practice.

## CASE REPORT

A 62‐year‐old woman presented to a local clinic with severe cough. She had a smoking history of 40 packs a year, but no notable past medical history or drug administration. Chest computed tomography revealed a large mass in the right upper lobe of her lung and enlarged lymph nodes in the right hilum.

She was referred to our department. Solid adenocarcinoma was detected by bronchial biopsy from the mass in the right upper lobe. The cancer was classified as stage IVB, with cT4N1M1c and isolated multiple brain metastases. Molecular testing of the biopsied specimen demonstrated KRAS G12C mutation. We performed gamma knife treatment for the brain metastases. Carboplatin, pemetrexed, and atezolizumab in combination were introduced as first‐line therapy. The primary lesion and right hilar lymph node metastases shrank rapidly.

**FIGURE 1:**
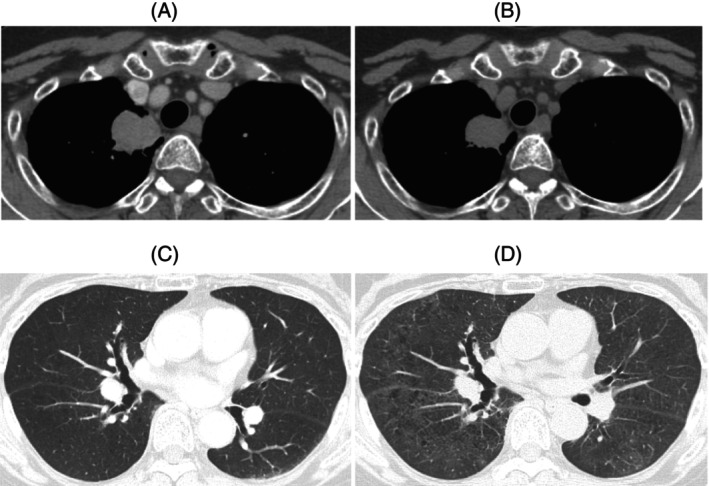
(A) Chest computed tomography before introduction of sotorasib reveals a mass shadow in the right upper lobe. (B) On day 80 after the start of sotorasib, the mass shadow had shrunk slightly. (C) Chest computed tomography before treatment with sotorasib shows no obvious abnormal shadows in the background lungs. (D) On day 80 after the administration of sotorasib, extensive ground glass opacities found in both lung lobes.

After four courses of initial therapy and 16 courses of maintenance therapy, we administered sotorasib (960 mg, daily) as second‐line therapy on day 79 of the last dose of atezolizumab because of re‐growth of the primary lesion. Although sotorasib led to stable disease, on the 80th day after starting sotorasib, cough and dyspnea appeared, and chest computed tomography revealed ground glass opacities in all lung lobes (Figure 1). The patient was admitted to our hospital. Bronchoalveolar lavage fluid showed an increased total cell count with lymphocyte predominance, whereas cytology and culture were both negative (Table 1). Cardiac ultrasonography demonstrated normal cardiac function and no evidence of heart failure. The patient was considered to have lung injury caused by atezolizumab or sotorasib.

**TABLE 1:** Findings of bronchoalveolar lavage fluid.

Bronchus	Rt.B^5^a
Recover rate	32.0%
Total cell count	3.06 × 10^5^/mL
Macrophages	77%
Neutrophils	2%
Lymphocytes	21%
Eosinophils	0%
Basophils	0%
Culture	Negative
Cytology	Class I

**FIGURE 2:**
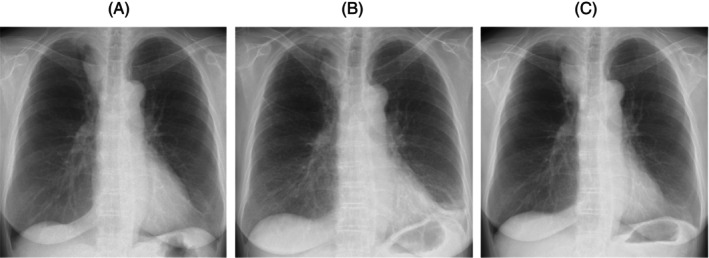
(A) Chest radiography before the introduction of sotorasib showing no ground glass opacities in both lung fields. (B) Chest radiography on day 80 after the start of sotorasib reveals ground glass opacities in both lung fields with decreased lung volume. (C) After prednisolone treatment, ground glass opacities in both lung fields disappear and lung volume improves, but the mass shadow in the right upper lung field increased.

**FIGURE 3:**
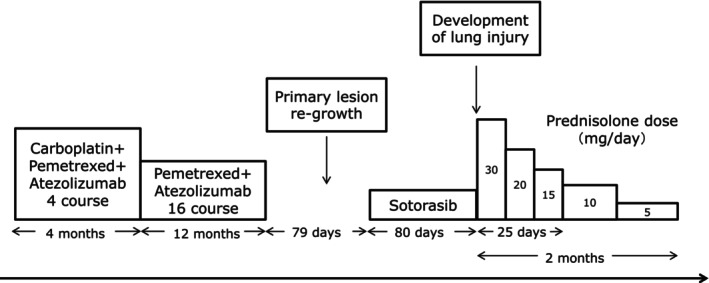
Clinical course of the present case.

Withdrawal of sotorasib did not improve the symptoms and shadows in both lungs. Prednisolone 30 mg/day (0.5 mg/kg/day) was introduced, and the lung disorder quickly resolved (Figure 2). One week after starting prednisolone therapy, percutaneous oxygen saturation in room air improved from 89% to 97%. The patient was discharged on the 16th hospital day. Tapering of prednisolone was performed as an outpatient. Prednisolone 15 mg/day or more was administered for 25 days, and treatment was completed in 2 months. She has not relapsed for several months following treatment (Figure 3).

The patient had a positive response without the need for high‐dose prednisolone and no lung injury recurrence despite early tapering of prednisolone, which are inconsistent with the treatment course observed in immune checkpoint inhibitor‐associated lung injury. However, since systemic corticosteroid is the standard therapy for drug‐induced lung injury of any cause, it was difficult to conclude with certainty that the lung injury was due to sotorasib based solely on the response to prednisolone alone.

## DISCUSSION

To the best of our knowledge, this is the first report describing in detail drug‐induced lung injury for which sotorasib was one of the suspect drugs. KRAS is a driver gene of cancer and the second most common mutation after EGFR in Japanese lung adenocarcinoma. The most common mutation subtype is G12C, accounting for about 30% of all KRAS mutations. Sotorasib is a molecular agent that targets the KRAS G12C mutation, and its efficacy was demonstrated in two clinical trials^1^, ^2^ for previously treated patients with advanced NSCLC harbouring KRAS G12C mutation. Although two cases of lung injury as an adverse event were reported in a previous study,^1^ it was unclear whether the lung injury was due to sotorasib since the patients had been treated with ICIs or chest radiation therapy.

If drug‐induced lung injury is suspected, infection or heart failure should be ruled out. When there are multiple suspected drugs, they can be withdrawn one at a time and the subsequent improvement in respiratory condition can be used to identify the drug responsible for the adverse events. Systemic corticosteroid therapy is indicated for patients that have no improvement after withdrawal and persistence of respiratory failure.

As the immune response by ICIs remains for several months after the end of treatment,^3^ we considered not only sotorasib but also atezolizumab to be suspect drugs. A previous retrospective study reported that prednisolone equivalent dose 15 mg/day for at least 4 weeks during corticosteroid tapering was linked to a reduced risk of immune‐related lung adverse event recurrence.^4^ In instances of immune‐mediated lung injury associated with respiratory failure, high‐dose prednisolone is often necessary as initial therapy. In the present case, the initial administration of prednisolone was sufficient to alleviate lung injury at a moderate dose, and the patient only required 25 days of prednisolone at 15 mg/day or higher and there was no recurrence of lung injury. Based on the treatment course observed, sotorasib was considered more likely than atezolizumab to have caused the lung injury. However, it was difficult to determine sotorasib‐induced lung injury because its response to prednisolone alone was not sufficient justification for identifying the suspect drug. It has been reported that immune‐related lung injury is associated with foamy macrophage accumulation in the alveoli and vacuolation of type II alveolar epithelial cells^5^; however, we were unable to confirm the presence of this phenomenon in the present case.

There have been reports of increased incidence of immune‐related adverse events, including lung injury, with osimertinib (third‐generation epidermal growth factor receptor‐tyrosine kinase inhibitor) after ICI treatment.^6^ Even though sotorasib is administered after ICIs, there have been no reports of lung injury caused by sotorasib. Further studies are needed to identify whether a history of ICI treatment prior to sotorasib administration poses a risk of lung injury.

It was important to distinguish whether ICIs or sotorasib induced the lung injury, because we considered a treatment strategy that included administration of sotorasib. As the clinical features, including onset time and typical imaging patterns, of sotorasib‐induced lung injury remain to be elucidated, accumulation of clinical cases is necessary in the future. Our case report is valuable as the first to identify drug‐induced lung injury following exposure to atezolizumab and sotorasib.

## AUTHOR CONTRIBUTIONS

Yuta Takahashi and Hiroaki Tachi wrote the manuscript. Ryo Watanabe and Kei Shimizu processed the image. Yusuke Yamamoto performed the manuscript review.

## CONFLICT OF INTEREST STATEMENT

None declared.

## ETHICS STATEMENT

The authors declare that appropriate informed consent was obtained for the publication of this manuscript and accompanying images.

## Data Availability

Data sharing is not applicable to this article as no new data were created or analyzed in this study.
